# Emotion Regulation, Effort and Fatigue: Complex Issues Worth Investigating

**DOI:** 10.3389/fpsyg.2022.742557

**Published:** 2022-02-16

**Authors:** Karol Lewczuk, Magdalena Wizła, Tomasz Oleksy, Mirosław Wyczesany

**Affiliations:** ^1^Institute of Psychology, Cardinal Stefan Wyszyński University, Warsaw, Poland; ^2^Department of Psychology, University of Warsaw, Warsaw, Poland; ^3^Institute of Psychology, Jagiellonian University, Kraków, Poland

**Keywords:** emotion regulation, effort, fatigue, self-control, mental fatigue, self-regulation

## Introduction

Emotion regulation (ER) refers to the processes by which individuals can influence the type of emotions they have, for how long as well as how strongly they experience and express them. ER is not simply constrained to diminishing negative emotions, but can include up- as well as down- regulation of both positive and negative emotional states, depending on one's regulatory aims (Koole, 2009; Gross, 2015; McRae and Gross, 2020). Moreover, emotion regulation can happen both on an individual level, as well as in a group context, and on a conscious and deliberate, as well as an automatic level (Braunstein et al., 2017; Nozaki and Mikolajczak, 2020; Porat et al., 2020).

The field of research on ER has grown rapidly, which is not surprising, if one takes into account the multitude of beneficial effects of efficient self-regulation of emotion on wellbeing, quality of social bonds, as well as education, learning and academic achievement (Gross and John, 2003; Graziano et al., 2007; Kraiss et al., 2020; Preece et al., 2021). Previous research points to the fact that, at least in some circumstances, ER requires significant effort to be exerted, which can affect its efficiency (e.g., Sheppes and Meiran, 2007; Webb et al., 2012; Stiller et al., 2019). This is especially the case for explicit and intentional ER of one's emotion (which is the focus of the current work) as automatic and implicit ER can potentially be applied with no significant effort (van Dellen et al., 2012). Investigating if and how ER effectiveness can diminish as a result of investing effort and the resulting fatigue is crucially important, as in daily life people are often put in circumstances that routinely require prolonged, incessant or recurring engagement in ER during work (see: emotional labor research, e.g., Choi and Kim, 2015; Yang and Chen, 2021), education and self-regulated learning (Ben-Eliyahu and Linnenbrink-Garcia, 2013) as well as social interactions (including interpersonal ER; Zaki and Williams, 2013). However, despite the practical importance of this issue, our understanding of the association between effort and fatigue on one side and ER on the other is still lacking, and is arguably one of the areas in which our knowledge on ER characteristics is still the most limited. The goal of the current manuscript is to provide a (1) critical summary of the previous research on the role of effort and fatigue in ER (including related research traditions, like mental fatigue and ego depletion paradigms), (2) identify the gaps in the current understanding of effortful characteristics of ER and (3) outline specific research recommendations, pertaining to underlying conceptualization, goals, as well as the methodology of studies connecting ER with effort and fatigue.

For the purposes of the current commentary, the literature search was performed in April 2021 using the following scientific databases: EBSCO, PubMed, and Google Scholar, using the following search terms: “emotion regulation,” “emotion control,” “emotion dysregulation,” “effort,” “fatigue.” Additionally, a number of articles were also included in the manuscript through cross-referencing.

## Self-Regulation, Self-Control and Effort

When discussing connections between ER and effort, evidence regarding effortful characteristics of other domains of self-control, e.g., cognitive control, should be taken into account. Traditionally, studies investigating effortful characteristics of self-control and changes in cognitive task performance mostly focused on the deterioration of performance over time, framed as a result of mental fatigue (Ackerman, 2010) or depleted resources (Baumeister et al., 2007).

### Mental Fatigue

Mental fatigue is a psychophysiological state of deterioration in the efficiency of mental control processes and reduced cognitive alertness that results in deterioration of task performance, connected with prolonged engagement in cognitively demanding activities (e.g., Rauch and Schmitt, 2009; Ackerman, 2010; Brietzke et al., 2021; Herlambang et al., 2021a). The longstanding line of research on mental fatigue investigates the effortful characteristics of mental activity and indicates that the effectiveness of control processes and executive functioning deteriorates during prolonged engagement in effortful self-regulation. In this research, the decreased efficiency of self-regulation is treated as an indicator of fatigue and effortful exertion of control processes. Previous studies have shown, for example, that accuracy in the Eriksen flanker task decreases gradually over a 2 h period (Faber et al., 2012) and in a go/nogo task, accuracy gradually decreases, while response time increases during 60 min of task engagement (Kato et al., 2009). In the data entry task, accuracy also decreases with prolonged engagement in the task (Healy et al., 2004). Also, performance in n-back tasks seems to decrease over a 120-min period (Shigihara et al., 2013).

As explicit ER also engages executive functions (Zelazo and Cunningham, 2007; Schmeichel and Tang, 2015; Pruessner et al., 2020), similar processes may also take place for ER and conclusions drawn from these studies are potentially important for posing hypotheses about ER and effort connections. However, as controlling emotional responses differs from control over cognitive processes in many aspects (the nature of the controlled stimuli is different; for emotional control, there is also a specific habituation process of the controlled stimuli. Finally, many forms of ER are more complex than tasks that require a subject to follow a simple behavioral pattern), there is still a need for research that will specifically address the effortfulness of explicit emotion control strategies. However, research has paid little attention to investigating the changes in the effectiveness of ER over time connected to exerting effort and causing fatigue. Presently, research on the effortfulness of ER is most often conducted using a “static” view of ER efficiency, where the effectiveness of ER during a particular period is averaged, and any fluctuations in its efficacy during this period are generally neglected (Webb et al., 2012). Moreover, in contrast to daily experiences, where we sometimes have to engage in longer or recurring episodes of ER and emotional coping, laboratory studies on ER tend to investigate relatively short, isolated episodes of ER (in most cases—a few minutes of ER, very rarely longer than 20 min) (Webb et al., 2012). This does not allow for capturing changes in ER efficiency that are related to exerting self-regulation effort over time and are, to some degree, analogous to changes that have been studied for cognitive control in a mental fatigue paradigm (Arai, 1912; Ackerman, 2010).

### Ego Depletion and ER Effortfulness

Aside from research on mental fatigue, another research branch that is of interest to effortful characteristics of ER is Baumeister's resource model of self-control (or *strength model*). Baumeister's model suggests that all forms of effortful and conscious self-control rely on a limited resource, which is depleted by engaging in acts of self-control (Baumeister et al., 2007). This depletion results in a state when our ability to self-control is diminished (called ego depletion). However, although valuable, conclusions about ER and effort that can be drawn from research on ego depletion may be limited by several factors (these factors are related to a possible future research directions, which are offered below, in the “Future research opportunities and directions” paragraph). First, experiments on ego depletion for emotional control have mostly focused on one emotion control strategy—suppression (Hagger et al., 2010). This actually stands in opposition to current emotion control theories that distinguish at least several effortful strategies (Koole, 2009; Gross, 2015; Naragon-Gainey et al., 2017; McRae and Gross, 2020). Secondly, in measuring the effectiveness of emotional control, ego depletion studies often relied on changes in the level of emotional expression (during such studies, participants' expression was typically videotaped and then appraised by judges), paying less attention to other channels of emotional responding (including changes in the subjective experience itself). It is also worth noting that in the studies based on a two task paradigm, emotional control in one task was always paired with control from another domain in another task (Hagger et al., 2010)—this makes interpretation of the results more complicated. Moreover, deterioration of self-control performance as an effect of exhausted resources is a key assumption of Baumeister's model, but within-task changes in the performance have never been tested within this paradigm (Hagger et al., 2010). Additionally, more recently, some assumptions and evidence of Baumeister's model have been contested from different angles, including an ongoing discussion on the motivational vs. physiological basis of the phenomenon (Kurzban, 2010; Inzlicht et al., 2014; Carter et al., 2015; Inzlicht and Friese, 2019; Dang et al., 2021; Vohs et al., 2021), which is further elaborated on in the next subsection. Moreover, poor replicability of the results in recent multi-lab preregistration projects raised additional concerns about the significance of the effect (Hagger et al., 2016; Lin et al., 2020; Englert and Bertrams, 2021). Interestingly, one possible explanation for these results is that although the effect of deterioration in self-control performance over time can be present, at least in some circumstances, the task duration (and possibly also effort intensity) used in the ego depletion paradigm (mostly <30 min) may simply be too short to reliably show the effects that are visible in the mental fatigue paradigm (in which longer tasks are usually used) (e.g., Hagger et al., 2010, 2016).

Irrespective of the results of the current ongoing discussion described above, the rich history of research on ego depletion offers significant insights into the factors affecting the effectiveness of self-control and self-regulation. At the same time, new theoretical and methodological approaches, which could enable researchers to investigate the relationships between ER, effort and fatigue, seem to be needed (see below for the possible future research directions).

### Psychological vs. Physiological Basis of Changes in Self-Control Efficiency Over Time

As described above, mental fatigue is described by experiential/subjective, behavioral, as well as physiological components. However, most studies include the assessment of only the first two, which makes it more difficult to derive a complete description of changes caused by mental fatigue (e.g., Van Cutsem et al., 2017). On a subjective level, mental fatigue is usually associated with elevated feelings of tiredness, weariness, lack of energy or exhaustion. At the same time, there are conflicting findings related to changes in motivation (Muraven and Slessareva, 2003; Marcora et al., 2009; Pageaux et al., 2014; Smith et al., 2015; Pires et al., 2018; Franco-Alvarenga et al., 2019) and mood alteration (van der Linden et al., 2003; Marcora et al., 2009; Gergelyfi et al., 2015; Herlambang et al., 2019, 2021a) due to mental fatigue (the role of motivation in mental fatigue is further discussed below). On a behavioral level, as discussed throughout this text, mental fatigue is usually associated with decreases in the performance effectiveness. However, the period needed to observe deterioration effects is usually longer (>30 min) than in ego depletion research (<30 min) (Hagger et al., 2016; Van Cutsem et al., 2017). On the physiological level, mental fatigue was most significantly associated with an increased theta and alpha band power in the frontal and prefrontal cortical areas, including slower activation in those brain regions (Wascher et al., 2014; Trejo et al., 2015; Arnau et al., 2021; Brietzke et al., 2021). Although indicators like heart rate variability, blood pressure and respiratory rate were studied in the context of mental fatigue (Richter et al., 2008; Hsu et al., 2015), it seems to be based on mechanisms that are rather separate from those that physical fatigue is usually characterized by, which originate in skeletal muscle activity (Dantzer et al., 2014; Abd-Elfattah et al., 2015). Moreover, the effects for theta and alpha band activity may not be as significant as shown in some previous studies, which corroborates the notion, as well as previous evidence, that pinpointing a physiological marker of mental fatigue is difficult (Dantzer et al., 2014; Abd-Elfattah et al., 2015; Brietzke et al., 2021) (also see the discussion on the role of glucose in effortful self-control as well as related research in the psychology of sports and performance: Carter et al., 2004; Chambers et al., 2009; Beedie and Lane, 2012; Molden et al., 2012; Sanders et al., 2012; Carter and McCullough, 2013; Hagger and Chatzisarantis, 2013; Finley et al., 2019). Due to this, subjective declarations of increased fatigue and tiredness as well as decreases in performance effectiveness are the main indicators of mental fatigue in previous research. This is in contrast to physical fatigue, for which physiological markers are more established (e.g., Rietjens et al., 2005; Mohanavelu et al., 2017; Polito et al., 2017) which allows for studying the fatigue processes in a way that is both based on an objective indicator, and not dependent on the performance effectiveness itself. Moreover, previous research has also shown that mental fatigue seems to limit subsequent performance in effortful physical tasks not through direct, measurable physiological mechanisms (e.g., increased heart rate or cardiac output), but through the psychological perception of greater effort, suggesting a predominant role of motivational, emotional, and attentional mechanisms; Marcora et al., 2009; Van Cutsem et al., 2017).

Due to this, many researchers point to the fact that deterioration in self-control effectiveness may be, in many cases, better explained by goal-related, motivational, and attentional shifts (that can occur during effortful self-control), than depleted physiological resources or other physiological changes (e.g., Inzlicht et al., 2014; Furley et al., 2019; Kelley et al., 2019; Hurley, 2021). Mental fatigue was also conceptualized as a consequence of cost/benefit calculations and opportunity cost analysis during effortful control (Kurzban et al., 2013) as well as a result of decreased motivation and goal activation (Herlambang et al., 2021b).

In line with this, previous research has shown that both physical and mental fatigue can be offset by increased motivation, i.e., a motivated agent can overcome the effects of fatigue, and sustain performance for a prolonged period of time (e.g., Hagger and Chatzisarantis, 2007; Li et al., 2011; Herlambang et al., 2019; Díaz-García et al., 2021). Particularly in the area of mental fatigue, previous research has shown that high intrinsic motivation counteracted some of the effects of mental fatigue, was related to better performance, investing higher effort as measured by both subjective and physiological indicators (pupillometry, heart rate variability) and being less distracted during task engagement (Herlambang et al., 2021a; see also: Hopstaken et al., 2015; Díaz-García et al., 2021). Another experimental study showed that for experimental conditions for which motivation was increased by additional reward, performance in a working memory task remained relatively stable for over 2 h, while performance decreased (showing mental fatigue effects) in the non-reward conditions (Herlambang et al., 2019). Some studies also showed that increasing motivation by offering monetary rewards for good performance can diminish mental fatigue effects (Boksem et al., 2006).

Similarly, previous studies have also shown that ego depletion can be moderated and/or mitigated by higher internal motivation or increased external motivation and additional incentives (Boucher and Kofos, 2012; Loschelder and Friese, 2016; Zhu et al., 2017). Within the sequential task paradigm, there is initial evidence that effortful control in which participants engage due to their own autonomous choice may not be as depleting as self-control which is not autonomously chosen (Moller et al., 2006). Further, previous research has indicated that effort mobilization effects, which increase performance in demanding cognitive tasks, can occur when achieving important goals involving the Self (Brehm and Self, 1989; Wright and Kirby, 2001; Gendolla and Richter, 2010; Gendolla et al., 2012). For emotion regulation, preliminary data show that participants can willingly engage in a more effortful form of ER under certain circumstances, e.g., when more effortful strategies offered higher disengagement from unpleasant stimuli (see: Sheppes and Levin, 2013). This invites another research question about the possible moderators and factors counteracting the changes in ER over time. Although ER is also a motivated process and can be significantly influenced by motivation (Tamir et al., 2020), there is not much evidence on how motivational and goal-related factors can impact fatigue and effort in ER—future studies could potentially bring interesting and important conclusions on this subject.

In line with the evidence reviewed above, as self-control in the domain of emotion—similarly to self-control in other domains—is also associated with the feelings of fatigue and effort (e.g., Sheppes and Meiran, 2007; Webb et al., 2012; Wong et al., 2017; Visser et al., 2018; Stiller et al., 2019) and is based on executive control processes (Zelazo and Cunningham, 2007; Schmeichel and Tang, 2015; Pruessner et al., 2020), it seems reasonable to study the effortful characteristics of ER in the context of the same motivational, attentional and goal-related processes. Following the proposed psychological models of ego-depletion and/or mental fatigue, prolonged engagement in effortful ER can potentially be accompanied by (1) shifting attentional focus, away from the cues signaling the need to restrain impulsive or habitual behavior, and toward the cues signaling gratification; (2) decreasing motivation for continual engagement in the effortful self-control, and—on the flip side—increasing motivation to behave impulsively or habitually (Inzlicht and Schmeichel, 2012; Inzlicht et al., 2014); (3) decreased activation of the long-term goals, in favor of the short-term ones (Herlambang et al., 2021b); (4) automatic cost-opportunity calculations leading one to diminish their investment in self-control exertion (Kurzban et al., 2013). These changes undermine one's ability to exert self-control with the same effectiveness and lead to decreases in performance quality. Described mechanisms also explain, why additional motivational incentives, which shift the motivational orientation back from impulsive behavior and toward restraint, can contribute to performance improvement after initial exertion.

Additionally, psychological and motivational frameworks are better at explaining the changes in self-control effectiveness that are more complex than just a gradual deterioration of performance. The available literature on emotion regulation training provides us with some evidence that ER employing more complex strategies (reinterpretation, distancing; Gross, 2015) can be improved with training and habit formation (Sheppes and Meiran, 2007; Denny, 2020; Moltrecht et al., 2021). It is possible that for complex forms of effortful ER, a period of performance optimization (before reaching its maximal effectiveness)—caused by learning and practice, cognitive warm-up, or similar effects—could possibly be observed, followed by a period of sustained efficacy, and finally, a period of performance deterioration. However, this hypothesis would have to be investigated in future studies, which are needed to answer theoretical and empirical questions on the relationship between ER and effort.

We also recognize that there may be substantial interindividual and intraindividual variability in the pattern, onset and magnitude of the changes in the ER effectiveness over time that are discussed in the current work. For example, feeling fatigue may be the cause of worsening performance for some individuals, but not others, depending on intrinsic beliefs, dispositional traits or motivational state. In line with this, previous studies showed that significant between-individual differences in ego depletion exist (Wenzel et al., 2019). Corresponding differences, pertaining to ER, should be investigated in future studies, following previous research investigating, the dispositional, as well as state and context related moderators of the ego depletion effect (Loschelder and Friese, 2016; Singh and Göritz, 2019; Wenzel et al., 2019).

### Disentangling ER Effort From ER Effectiveness in Recent Studies

As mentioned above, in many studies, including mental fatigue research, a decrease of self-regulation efficiency over time is used as an indicator of the level of effortfulness that a specific self-regulation task requires or the presence of fatigue (Rauch and Schmitt, 2009; Ackerman, 2010; Faber et al., 2012). However, there is now increasing evidence that other indicators can be used as a proxy for effort. Moreover, these indicators can differentiate between subjects and groups even when the sheer efficiency of ER is the same (e.g., Soto et al., 2016). Therefore they can be used as a valuable source of independent and unique information about effort and fatigue effects in ER, alongside changes in ER efficiency itself. These indicators include, for example, a stimulus proceeding negativity (EEG evoked potential, Moser et al., 2017), pupil dilation (Strauss et al., 2016; Scheffel et al., 2021), the activity of particular brain regions detected in fMRI (e.g., Dörfel et al., 2014; Ellard et al., 2017), as well as fNIRS studies (Lu et al., 2019; Azhari et al., 2020), but also subjective self-reports of effort and fatigue (Wong et al., 2017; Visser et al., 2018). Interestingly, digital biomarkers of mental fatigue, like smartphone-based gaze detection, are also being developed (Tseng et al., 2021). Discussed indicators of effort, along with examples of their use and main results, are presented in Table 1 (the table does not aim to provide a systematic review of research on ER effort indicators, rather it describes the most popular and promising indicators and provides examples of their use in previous research on fatigue and effort).

**Table 1:** Indicators of emotion regulation effort (other than changes in sheer ER effectiveness) in available studies—examples of use.

**Indicator**	**Methodology**	**Main results**	**Examples of research and additional comments**
Subjective declaration of fatigue and/or effort invested in emotion regulation	Self-report	Self-report measures include, for example, single items, regarding the invested effort, asked after the task, for example: *How much effort did you invest to manage your emotions during this social interaction?* (answers given on a scale from 1 [*little effor*t] to 5 [*a lot of effort*]). The main flaw of this index is its retrospective character and lack of possibility of tracking the dynamics during the ER task. Self-report measures are also used in ecological momentary assessment as, for example, retrospective measures at the end of the day; they have proven to enable the detection and evaluation of ER abnormalities in clinical populations.	Wong et al., 2017; Visser et al., 2018
Stimulus proceeding negativity	Electroencephalography (EEG, evoked potentials)	Event-related potential (ERP) is the brain's electrophysiological response to a stimulus. The amplification or attenuation of specific ERPs can be indicative of the amount of effort employed in ER. For example, stimulus preceding negativity is an ERP marker of self-control and its amplification can be used to determine whether self-referential emotional reactivity reduction employed effort. On the other hand, another ERP—the late positive potential has been consistently shown to decrease when effortful ER strategy was employed.	Moser et al., 2017
Pupil dilation	Pupillometry	Changes in pupil dilation are the involuntary signs of the autonomic nervous system's response. They reflect both cognitive effort, as well as emotional arousal. Pupil dilation is a reliable source of information about ER effortfulness and can distinguish between specific emotion regulation strategies in terms of their effortfulness. It was also proven useful in differentiating in the extent of cognitive effort employed in ER across different group ages. Also, pupil dilation decreasing with time can indicate the habituation process.	Strauss et al., 2016; Martins et al., 2018; Langer et al., 2021; Scheffel et al., 2021
Activity of specific brain regions	Functional magnetic resonance imaging (fMRI)	Effortful ER has been consistently linked to the activity of prefrontal-parietal structures associated with cognitive control. The most commonly reported structures are the ventrolateral and dorsolateral prefrontal cortex. Effortful ER also involves the dorsomedial prefrontal cortex and posterior parietal cortex, anterior cingulate cortex and inferior parietal cortex. Researchers also highlight the importance of amygdala-frontal functional connectivity in ER (studied in the resting-state paradigm). fMRI allows clinical populations to be distinguished from healthy controls, however, further examination is needed, for example, to disentangle the differences in neural activity for different ER strategies.	Banks et al., 2007; Dörfel et al., 2014; McLaughlin et al., 2015; Xie et al., 2016; Moser et al., 2017; Zhang et al., 2020. For a meta-analysis of research on brain activity during cognitive reappraisal, see: Buhle et al., 2014
	Functional near-infrared spectroscopy (fNIRS)	This methodology allows for studying the changes in the concentration of oxygenated hemoglobin (O2Hb) and deoxygenated hemoglobin are detected with the aid of near-infrared rays. The increase in (O2Hb) in specific brain regions is an indicator of energy consumption and reflects the greater effort. This increased energy consumption in specific brain regions. fNIRS has been, for example, used in studies distinguishing between effort exerted in surface and deep acting or to check whether effort invested in ER is dependent on context.	Lu et al., 2019; Azhari et al., 2020
Heart rate	Electrocardiography (ECG)	Increased heart rate reflects a greater autonomic response and was used as an indicator of greater effort and differentiated between reappraisal and acceptance strategies; reappraisal was more effective, but also more effortful yielding a greater autonomic response.	Goldin et al., 2019
Heart rate variability (HRV)	Electrocardiography (ECG)	Heart rate variability is the indicator of the variability of the duration of the periods between consecutive heartbeats. Increased HRV was proven to reflect decreased cardiovascular effort. After ER training the ER task elicited increased HRV. HRV was also used as a measure of ER effectiveness itself, and not its effortfulness. Disentangling physiological changes that happen as a result of emotional arousal (indicating ER failure) vs. as a result of effort exerted in ER can be difficult. Therefore, aside from using indicators of effort like HRV and skin conductance (see below), it is still reasonable to use sheer changes in ER efficiency over time as an indicator of fatigue and effortful exertion of self-regulation processes (following mental fatigue research) as well as a multitude of sources (multiple indicators) to gather full information about effort and fatigue involved in ER processes.	Christou-Champi et al., 2015 For HRV as a measure of ER effectiveness see also: Denson et al., 2011
Pre-ejection period (PEP)	Electrocardiography (ECG)	The pre-ejection period is defined as the period between the onset of left ventricular contraction and aortic valve opening and reflects the response of the sympathetic nervous system. Greater task engagement (due to e.g., its difficulty) was associated with a greater sympathetic response which was reflected by a proportional shortening of PEP. On the contrary, better ER regulation abilities were linked to PEP lengthening.	Gendolla and Silvestrini, 2010; Kahle et al., 2016
Skin conductance level (SCL)	Electrodermal activity (EDA)	Skin conductance level informs about the activity of sweat glands at the surface of the skin. Increased SCL indicates increased arousal that may reflect greater physical or psychological effort invested in ER. Differences in SCL between people of different cultural backgrounds may suggest differences in cognitive and psychological effort invested in the suppression of emotions; skin conductance levels may be treated as an indicator of ER effortfulness.	Soto et al., 2016 Just as HRV, skin conductance level was also used as a measure of ER effectiveness, rather than effort: Opitz et al., 2014

## Future Research Opportunities and Directions

A short review of the studies provided above illustrates that it would be beneficial to further study the effortful characteristics of ER. Below, we outline several research opportunities and directions that may further improve our state of knowledge on how effort and fatigue influence ER effectiveness.

(1) To inspect the effects of effort and fatigue on ER effectiveness, it would be beneficial for studies to adopt **a dynamic view**, in which within-episode (or within-task) changes in ER effectiveness are analyzed, based on studies employing longer episodes of ER (over 20 min). This would allow for the possibility of uncovering changes in ER effectiveness due to effort and/or fatigue, that would not be possible to find within shorter episodes of ER. Engagement in prolonged ER can also cause habituation effects (the onset and magnitude of habituation for emotional stimuli during intentional ER is an interesting research question in itself). The effects of habituation, however, can be controlled to a significant degree and/or counteracted. Many studies indicate that emotional habituation takes place after repeated exposure to the same stimuli, especially when the set of stimuli is small (e.g., Siddle, 1991; Feinstein et al., 2002). Using a large set of diverse stimuli may therefore help counteract the habituation effect. Additionally, in some studies (e.g., Denny and Ochsner, 2014), varied trial length prevents participants from forming firm expectations about the onset of emotional stimuli for which emotional response needs to be regulated (this can increase their alertness and possibly decrease habituation effects). Lastly, using a control group that would be exposed to the same experimental procedures and emotional stimuli, but at the same time would not be instructed to control their emotions (but to experience and express them naturally), may help in differentiating the effects of sheer habituation (control group) vs. habituation and effortful emotion control (experimental group).
(2) A diverse set of independent indicators pertaining to the effort exerted during ER other than sheer ER effectiveness could potentially be employed in future studies (see Table 1 for more information).
(3) Studies on ER and effort may benefit from taking into account the most important **moderators of ER effectiveness**. Three kinds of factors influencing ER efficacy have been identified so far: those related to the characteristics of regulated stimulus (e.g., valence and strength of regulated emotions), to characteristics of the controlling agent (e.g., personality traits, ER abilities), and to specific ways the agent controls the stimulus (used regulation strategy) (Gross and John, 2003; Webb et al., 2012; Gross, 2015; Kobylińska and Kusev, 2019). In the future, research can be aimed at investigating how the effortfulness of ER changes depending on these factors.
(4) Moreover, investigating the effortfulness of ER strategies based on **different components of emotion** may lead to important results., i.e., different measurement levels: expression, experience, physiology and neurophysiology. The effectiveness of self-control for different components of the emotional response is often not unitary, and in some cases engaging in self-control can have opposite effects depending on the measured component of emotion (Webb et al., 2012; Mohammed et al., 2021). This is illustrated by the fact that, as indicated by previous research, emotional suppression can be effective in controlling expression/behavior, ineffective on an experiential level and counter-effective on a physiological level within the same episode of ER (Webb et al., 2012). This shows the benefit of including various levels of measurement (emotion components) in the research—each of these levels provides us with unique information about the course of emotion control and each is equally important. Such research would be greatly facilitated by the fact that established indicators of ER effectiveness on each of these levels are available, for example: (1) Heart Rate Variability on a physiological level (Appelhans and Luecken, 2006; Thayer and Lane, 2009; Denson et al., 2011; Witvliet et al., 2011), (2) specific face muscle activity as measured with electromyography (EMG) as an indicator of the strength of emotional expression (Ray et al., 2010; Witvliet et al., 2011). On the neurophysiological level evoked potentials like Late Positive Potential (LPP; MacNamara et al., 2009; Thiruchselvam et al., 2011; Hajcak et al., 2014) have proven to be useful in previous research.

### Other Areas for Future Studies

There is also a wide body of research regarding ER effort in subclinical and clinical populations pointing to the importance of exploration and understanding ER deficits. For example, previous studies have shown that some clinical populations (e.g., maltreated adolescents) may not differ from general population control groups in ER effectiveness, but may have to employ greater effort in ER (McLaughlin et al., 2015). Increased activation of brain regions involved in effortful ER was found for at least several psychiatric disorders characterized by ER deficiencies [e.g., borderline PD, schizophrenia (Zhang et al., 2020); suicidal ideation (Miller et al., 2018)]. Further research regarding clinical populations would be helpful in explaining the role of ER effort and its dynamics in the development and maintenance of these disorders. Such knowledge can be a significant aid in developing well-adjusted treatment strategies, especially for conditions for which general emotion dysregulation or lack of ER flexibility is a central characteristic (e.g., Conroy et al., 2020). Additionally, one of the most interesting directions for future research seems to be investigating emotion regulation effort in the social context, i.e., interpersonal emotion regulation (Rimé, 2009; Zaki and Williams, 2013) or intrapersonal emotion regulation which happens directly in a social context (Wong et al., 2017). As some of the markers of effortful self-control are hard to gather outside of the laboratory setting (see Table 1), studying effort involved in ER in the social context is often based on self-reported evaluations of ER effort during a particular social interaction (e.g., Visser et al., 2018). Such studies brought evidence that, for example, higher reported ER effort predicts lower wellbeing after social interaction, which can be buffered by the attainment of the regulatory goal (Wong et al., 2017). Future studies on effort involved in ER in the social context may benefit from employing objective indicators of effort—which would optimally be gathered amidst social interactions—instead of declarative, retrospective measures. Studying the qualities of ER in the social context, including its effortfulness, is also of importance for clinical populations as, for example, interpersonal ER is argued to be the underlying mechanism of social support in depression (see: Marroquín, 2011).

## Conclusions

Although previous studies have offered very valuable knowledge, they have not yet provided a comprehensive picture of how ER effectiveness can change due to effort and fatigue. Such changes were successfully studied for other domains of self-regulation within the mental fatigue paradigm. Research on ER can draw upon this research and further investigate this subject by (1) employing longer episodes of ER (which would be consistent with daily life) and investigating dynamic changes in ER effectiveness over time (2) studying the obtained effects for different components of emotion (experience, expression, physiology), (3) different ER strategies and (4) for the emotion of different valence, type and intensity. Such research can provide important information on effort and fatigue related to ER, which is currently lacking.

## Author Contributions

KL was responsible for the conceptualization, structure of the article, and was responsible for funding acquisition. KL and MWi conducted literature review and wrote the first draft of the manuscript. TO and MWy provided feedback and critical review of the first draft of the manuscript. All authors contributed to and have approved the final manuscript.

## Funding

The preparation of this manuscript was supported by Preludiun grant awarded by National Science Centre, Poland to KL, grant number: 2016/21/N/HS6/02678.

## Conflict of Interest

The authors declare that the research was conducted in the absence of any commercial or financial relationships that could be construed as a potential conflict of interest.

## Publisher's Note

All claims expressed in this article are solely those of the authors and do not necessarily represent those of their affiliated organizations, or those of the publisher, the editors and the reviewers. Any product that may be evaluated in this article, or claim that may be made by its manufacturer, is not guaranteed or endorsed by the publisher.
